# Microstructural Study of a Mg–Zn–Zr Alloy Hot Compressed at a High Strain Rate

**DOI:** 10.3390/ma13102348

**Published:** 2020-05-20

**Authors:** Jing You, Yingjie Huang, Chuming Liu, Hongyi Zhan, Lixin Huang, Guang Zeng

**Affiliations:** 1 School of Materials Science and Engineering, Central South University, Changsha 410083, China; Jing_Y@csu.edu.cn (J.Y.); alex.huang@csu.edu.cn (Y.H.); cmliu@csu.edu.cn (C.L.); 2 China Science Lab, General Motors Global Research and Development, Shanghai 201206, China; 3 CITIC Dicastal Co., Ltd., Qinhuangdao 066000, China; huanglixin@dicastal.com

**Keywords:** Mg–Zn–Zr alloys, EBSD, hot compression, dynamic recrystallization, bimodal microstructure

## Abstract

Understanding the correlation of plasticity with deformation and dynamic recrystallization (DRX) behaviors, in magnesium (Mg) alloys deformed under high-strain-rate conditions, is increasingly important for wrought Mg processing. In the present study, a ZK30 (Mg-2.61%Zn-0.66%Zr by weight percent (wt.%)) alloy in the as-forged state was hot compressed to various strain levels at a temperature of 350 °C and a strain rate of 10 s^−1^. Heterogeneous deformation and dynamic recrystallization (DRX) behaviors of the complicated microstructures in the deformed samples were analyzed via a grain-partitioning approach based on intra-grain misorientation analysis from electron back-scattered diffraction (EBSD). The ZK30 alloy showed excellent formability, remaining intact at a true strain of −1.11. Continuous dynamic recrystallization (CDRX) and discontinuous dynamic recrystallization (DDRX) via grain boundary corrugation/bulging are the dominant mechanisms for the relaxation of strain energy during hot compression. Initial Zr-rich coarse grains undertook a significant portion of the plastic strain as the compression progressed, reflected by the increased misorientations within their interior and marked change in their aspect ratios. The results indicate that the excellent plasticity of the as-forged ZK30 alloy can be attributed to the operative CDRX mechanisms and the reduced deformation anisotropy of Zr-rich coarse grains containing Zn–Zr nano–precipitates.

## 1. Introduction

Magnesium (Mg) alloys, due to their low densities and high specific strength, are attractive for lightweight applications, catering to the demand of the automobile industry for reduced emissions and improved fuel economy [1]. However, the formability of hexagonal-close-packed (HCP) metals in plastic deformations is deficient due to inadequate independent slip systems [2]. Therefore, most of the Mg components in vehicles are made by casting, especially high-pressure die casting, to take advantage of the castability and low melting points of Mg alloys [3].

Referring to previous studies [4,5,6,7,8,9], Mg wrought alloys are generally suitable for processing at temperatures higher than 300 °C and strain rates lower than 1 s^−1^ to avoid cracking. At higher temperatures and lower strain rates, non-basal slip systems can be readily activated to accommodate strains, and dynamic recrystallization (DRX) can operate for relaxation of stress concentrations. However, processing at elevated temperatures and low strain rates is energy consuming and unfavorable to productivity, which induces high costs. Employment of high strain rates in the hot deformation of Mg alloys may be a potential solution for cost reduction. Moreover, a higher strain rate will contribute to finer DRXed grains in the final microstructure, which will enhance mechanical properties. However, it is challenging to deform Mg alloys to a large strain level at strain rates higher than 1 s^−1^ during hot deformation. A common phenomenon related to cracking and failure is severe shear localization occurring during deformation at a high strain rate [4,6,10]. There are two mainstream explanations for the formation of shear bands in Mg alloys. The first is that compression along the c-axes of grains tends to activate compression/double twinning in the basal-textured Mg samples. High activity of basal slip and repetitive DRX will occur within the twins since their orientations are more geometrically preferable for basal slip comparing with the basal-textured matrix [7,11,12,13,14]. This contributes to strain localization and the formation of macro-shear-bands (macro-SBs). An alternative explanation is to correlate shear localization with DRXed grains whose orientations are randomized from the basal texture. A ‘necklace structure’ consisting of DRXed grains of random orientations formed around the coarse-grained matrix of basal texture favors basal slip. Hence, more plastic strains will partition into the ‘necklace structure’ to form shear bands [15,16]. Since a high strain rate will enhance twinning activities and the sensitivity of basal slip to grain orientation, Mg alloys are more vulnerable to shear localization when being hot deformed at high strain rates. Moreover, DRX kinetics are lower under high-strain-rate conditions.

In recent years, Zhu et al. [17] reported that high-strain-rate rolling can be performed on the ZK60 Mg alloy at an equivalent strain rate as high as ~9.6 s^−1^ at 300 °C. An intact sheet was successfully produced. The authors attributed the excellent plasticity of the ZK60 alloy to the twinning-induced DRX (TDRX) mechanism [18,19] which effectively released strain energy. It is worth noting that the as-received material used for hot rolling/compression in their experiments was in the as-cast state and had an average grain size of ~150 μm. This coarse-grained microstructure is beneficial for the operation of TDRX. However, in industrial production, the as-received materials are usually extruded, hot rolled or forged rather than cast alloys. Mg alloys after plastic deformation processing have a much finer grain size. In general, a fine grain size will suppress the activation of twinning [20]. In this case, the deformation and DRX behaviors of Mg alloys of fine grain size in the hot deformation at high strain rates remain unknown and require further studies.

It is industrially important to manufacture wrought Mg components by hot deformation (over 300 °C) with a high production rate. To explore this feasibility, a ZK30 (Mg–2.61%Zn–0.66%Zr by wt.%) alloy in the as-forged state was hot compressed at a high strain rate of 10 s^−1^ at 350 °C to various strain levels in this study. The initial grain size in the microstructure of the as-forged ZK30 alloy was refined compared to the as-cast microstructure. For industrial production, it is not feasible to apply strain rates much higher than 10 s^−1^ due to a concern over safety and operability. Therefore, the testing strain rate in the present study was set to 10 s^−1^. In general, Mg alloys will be heated to over 300 °C prior to plastic forming. Therefore, the testing temperature in the present study was set to 350 °C. Systematic microstructural characterizations by correlative optical microscopy (OM) and electron backscattering diffraction (EBSD) were conducted on the hot-compressed samples. The correlation of plasticity with deformation and DRX behaviors of the as-forged ZK30 alloy under the present deformation condition is discussed.

## 2. Experimental Procedures

**Figure 1:**
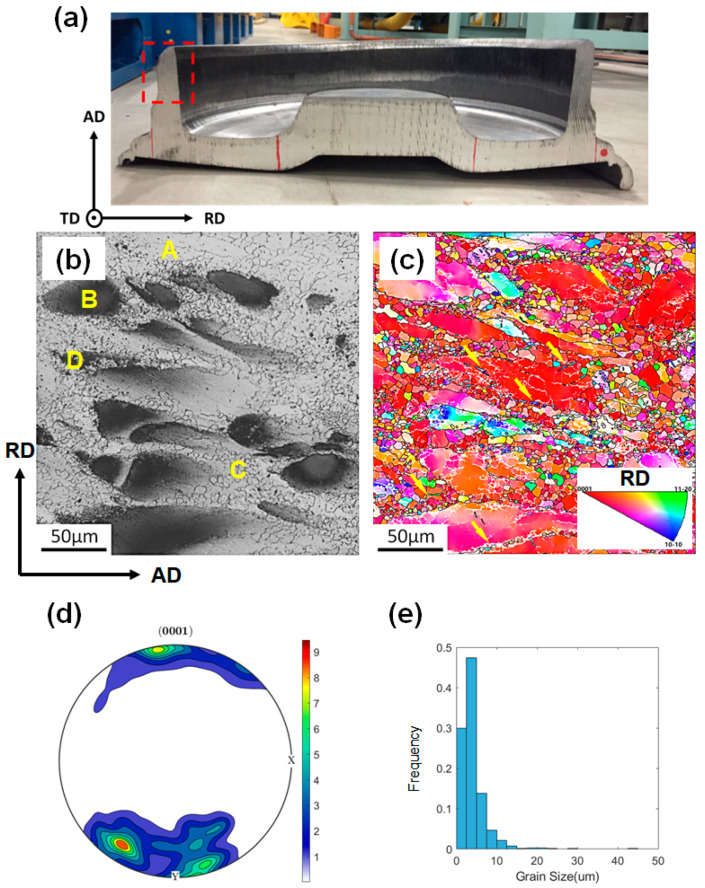
Initial microstructure of the as-received sample: (**a**) location of sampling for hot compression, (**b**,**c**) corresponding optical microscope (OM) and electron back-scattered diffraction (EBSD) micrographs of (**a**), (**d**) corresponding (0001) pole figure, and (**e**) grain size distribution calculated from the EBSD dataset of (**c**). The step size for EBSD measurements was 0.3 μm.

A ZK30 (Mg–2.61%Zn–0.66%Zr by wt.%) forged blank formed at 390 °C, as shown in Figure 1a, was supplied by CITIC Dicastal Co., Ltd. (Qinhuangdao, Hebei, China), for study. Here, the axis direction, radial direction, and tangential direction of the forged blank are referred to as AD, RD, and TD, respectively. Cylindrical samples with a dimension of Ø10 mm × 12 mm were cut from the rim with their long axes aligned along the RD for hot compression tests. Uniaxial hot compression tests were conducted on a Gleeble-3180 thermal–mechanical simulation testing system. Nickel-based lubricants and graphite sheets were used on both ends of the sample to reduce friction. Before the tests, two thermocouples were welded on the surface of the sample for temperature control. Then, the sample was heated at a heating rate of 5 °C/s to 350 °C and held for 120 s to ensure uniform temperature distribution. The sample was uniaxially compressed at 350 °C and 10 s^−1^ followed by water quenching. Since direct quenching in the chamber is not allowed, there was an interval of approximately 4–5 s between the end of compression and the water quench. The true strains (ε_t_) of compression tests at a strain rate ($\dot{\varepsilon }$) of 10 s^−1^ were set to −0.22, −0.40, −0.69, and −1.11, i.e., height reductions of 0.2, 0.33, 0.5, and 0.67, respectively. For comparison, compression tests at 350 °C and 10^−3^ s^−1^ were conducted following the same procedure, with ε_t_ of −0.40 and −1.11. Measurements were repeated 2–3 times for each condition to ensure the reproducibility of stress–strain curves.

The microstructures of tested samples were characterized using a Leica DIM5000 optical microscope (OM) (Wetzlar, Germany) and a Zeiss AURIGA field-emission gun scanning electron microscope (SEM) equipped with an EBSD detector (Zeiss, Heidenheim, Germany). The samples were cross sectioned transversely along the centerline and then ground and polished following standard procedures. Samples for OM and SEM observations were etched by a solution of 0.5 wt.% oxalic acid, 0.5 vol.% nitric acid, and 0.5 vol.% acetic acid for 15 s. Electrochemical polishing was performed on polished samples for EBSD characterization, using a solution of 3 vol.% perchlorate and 1 vol.% nitric acid ethanol at −40 °C for 90 s. The step size applied in the EBSD characterization was between 0.12–0.3 μm, depending on the grain size of the detected samples. EBSD datasets were analyzed using MATLAB™ 9.2 (Mathworks, Natick, MA, USA) with the MTEX 5.28 toolbox [21]. Transmission electron microscopy (TEM) samples were collected by the focused ion beam (FIB) lift-out technique using an FEI Helios NanoLab 600 dual beam system. TEM characterization was performed on a Titan G2 60–300 microscope at 300 kV (FEI, Hillsboro, OR, USA).

## 3. Results

### 3.1. As-Forged Microstructure

The as-received material revealed a typical partially DRXed microstructure with bimodal features, as shown in Figure 1b. Most coarse grains were in dark contrast, which is caused by the dispersion of the refined Zn–Zr phase [22,23,24]. Zr particles, which acted as nucleation substrates for α-Mg, were dissolved into the Mg matrix during solidification. Hence, the Zn–Zr phase precipitated out from the as-cast Mg dendrite supersaturated in Zr in the subsequent annealing process [25]. The pinning force exerted on mobile dislocations by Zn–Zr precipitates retarded the movement of dislocations, leading to the partial DRX behavior of ZK30 during forging. The as-forged microstructure can be divided into four types of regions: the Zr-lean coarse-grained region (marked by ‘A’ in Figure 1b), the Zr-rich coarse-grained region (marked by ‘B’ in Figure 1b), the Zr-lean fine-grained region (marked by ‘C’ in Figure 1b), and the Zr-rich fine-grained region (marked by ‘D’ in Figure 1b). Coarse grains were surrounded by fine DRXed grains. Figure 1c shows the inverse pole figure (IPF) map of the same region as Figure 1b. A small quantity of twin-like features can be observed as indicated by yellow arrows. The corresponding pole figure in Figure 1d reveals that most grains tended to align their c-axes to the RD. In addition, it is noted that the maximum intensity of the basal pole was slightly inclined to the AD. The grain size distribution is shown in Figure 1e. Nearly 80% of grains were smaller than 5 μm in the as-forged microstructures.

### 3.2. OM Characterization of the Compressed Microstructures

**Figure 2:**
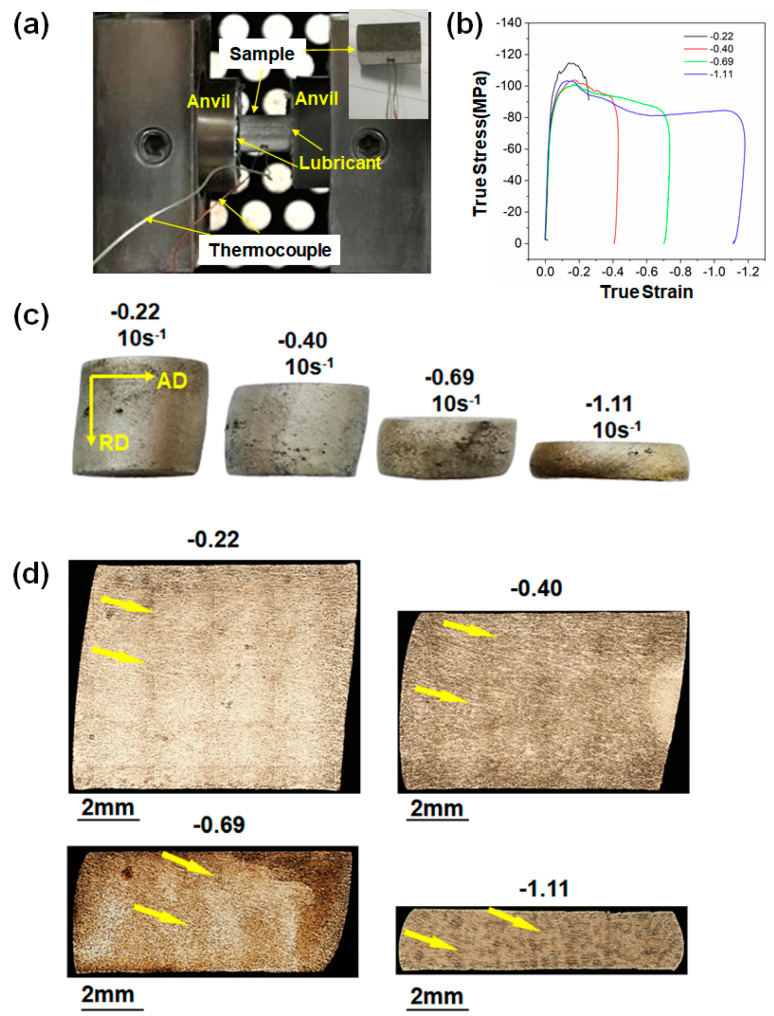
Interrupted hot compression tests of ZK30 samples conducted at 350 °C and $\dot{\varepsilon }$ of 10 s^−1^: (**a**) experiments setup in the Gleeble system, (**b**) compressive true strain–stress curves with final strains of −0.22,−0.40, −0.69, and −1.11, (**c**) pictures of ZK30 cylinders compressed to ε_t_ of −0.22, −0.40, −0.69, −1.11, and (**d**) corresponding macro-structures with yellow arrows marking the boundaries of shear bands (the samples were cross sectioned transversely along the centerline).

**Figure 3:**
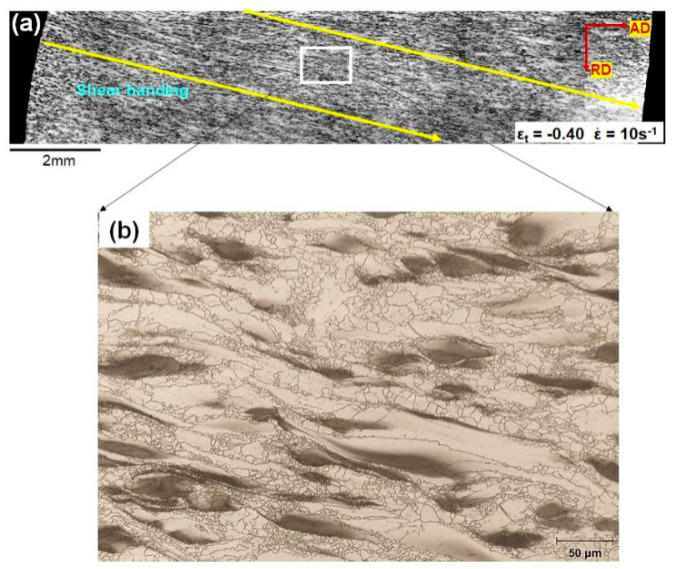
Optical micrographs of macro-SBs at ε_t_ of −0.40 under the high-strain-rate condition: (**a**) overall microstructure at a lower magnification, (**b**) microstructure in the framed region of (**a**) at a higher magnification.

Uniaxial hot compressions were performed on cylindrical samples with the loading axis parallel to the RD as shown in Figure 2a. Figure 2b provides the compressive true stress–strain curves of ZK30 samples hot compressed at $\dot{\varepsilon }$ of 10 s^−1^ at 350 °C. Strain hardening continued until the peak stress reached >100 MPa at ε_t_ of ~0.17. Stress gradually declined beyond the peak due to DRX softening. The tested samples remained intact up to ε_t_ of −1.11 as shown in Figure 2c, showing excellent plasticity under the present deformation condition. It is noted that all the samples showed anisotropic flow with the major axis of the cross-section developed toward the AD. For consistency, all the deformed samples were observed on the AD–RD section viewed along the TD. Optical images in Figure 2d indicate that macro-SBs (as indicated by yellow arrows) developed early at ε_t_ of −0.22 with a weak contrast. The contrast of macro-SBs increased with increasing strains. Optical images of macro-SBs (as delineated by yellow arrows) at higher magnifications are shown in Figure 3. It is evident that the contrast of macro-SBs is due to the significant shearing of Zr-rich coarse grains.

**Figure 4:**
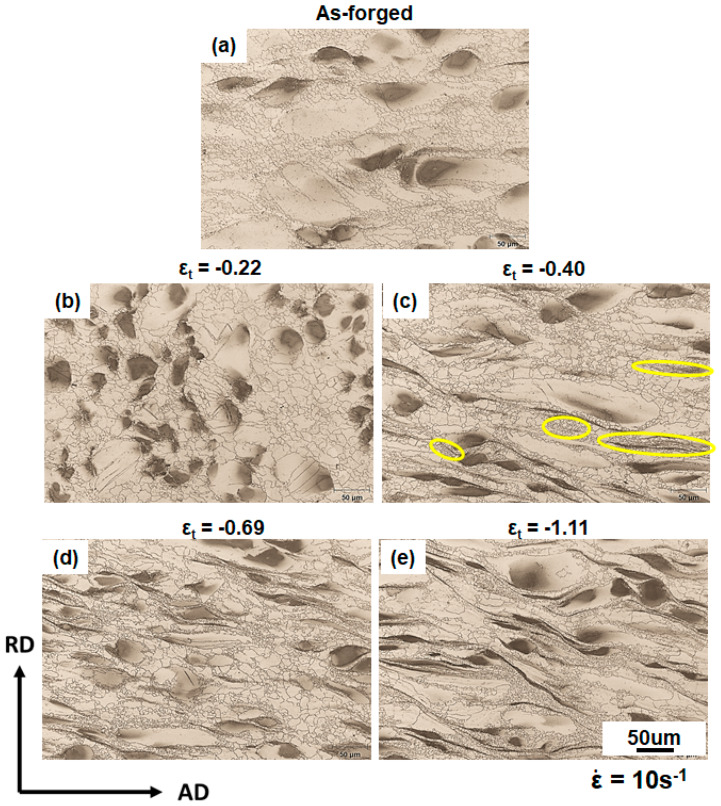
Optical micrograph of microstructures after compression with ε_t_ of (**a**) 0, (**b**) −0.22, (**c**) −0.40 (**d**) −0.69, and (**e**) −1.11 at 350 °C and 10 s^−1^. Yellow ellipses highlight refined DRXed grains formed near the Zr-rich coarse grains.

Figure 4 shows the detailed microstructural evolution of ZK30 alloys hot deformed to various strains at $\dot{\varepsilon }$ of 10 s^−1^. The characterizations were conducted on the central location of macro-SBs. Though the strain rate employed was as high as 10 s^−1^, few twinning signatures were observed. Only a small quantity of twin-like features existed within coarse grains. Twinning activity was suppressed due to the synergic effects of the refined grain size and elevated testing temperature. At ε_t_ of −0.22, it was surprising to find that extensive growth of Zr-lean equiaxed grains occurred (Figure 4b) in comparison to the as-received material (Figure 4a). Most coarse grains did not show a sensible morphological change. As ε_t_ increased to −0.40, the aspect ratios of coarse grains noticeably increased (Figure 4c). As indicated by yellow ellipses in Figure 4c, DRXed grains nucleated around the Zr-rich coarse grains, which were finer than the initial DRXed grains in the as-received microstructure. As the deformation proceeded to ε_t_ of −0.69 and −1.11, the fraction of refined DRXed grains increased and the aspect ratio of coarse grains was significantly altered.

**Figure 5:**
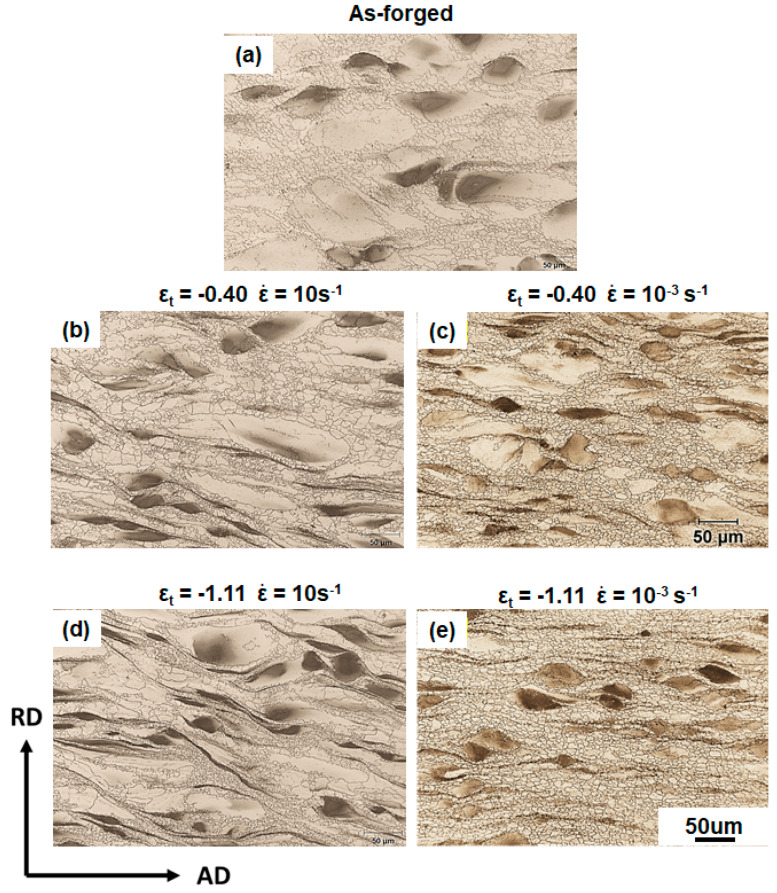
Optical micrographs of microstructures in different states: (**a**) as-forged state, after deformation at $\dot{\varepsilon }$ of 10 s^−1^ with ε_t_ of (**b**) −0.40 and (**d**) −1.11, after deformation at $\dot{\varepsilon }$ of 10^−3^ s^−1^ with ε_t_ of (**c**) −0.40 and (**e**) −1.11.

Figure 5 shows the comparison of the microstructures of the samples hot compressed to ε_t_ of −0.40 and −1.11 at different strain rates of 10 s^−1^ and 10^−3^ s^−1^, respectively. Distinct deformation behaviors influenced by strain rates were implied from the microstructural observations. Different from the sample deformed to ε_t_ of −0.40 at $\dot{\varepsilon }$ of 10 s^−1^ (Figure 5b), the sample deformed to the same strain level at $\dot{\varepsilon }$ of 10^−3^ s^−1^ showed a relatively homogeneous grain size distribution, and no abnormal grain growth was observed (Figure 5c). In addition, at ε_t_ of −1.11, nearly all the coarse grains were consumed by DRXed grains under the quasi-static condition (Figure 5e), whereas a certain fraction of the Zr-lean coarse grains remained under the high-strain-rate condition (Figure 5d). This is likely due to the fact that DRX kinetics decrease with increasing strain rate.

### 3.3. EBSD Characterizations of the Compressed Microstructures

#### 3.3.1. Grain Average Misorientation Analysis of Global Microstructures

**Figure 6:**
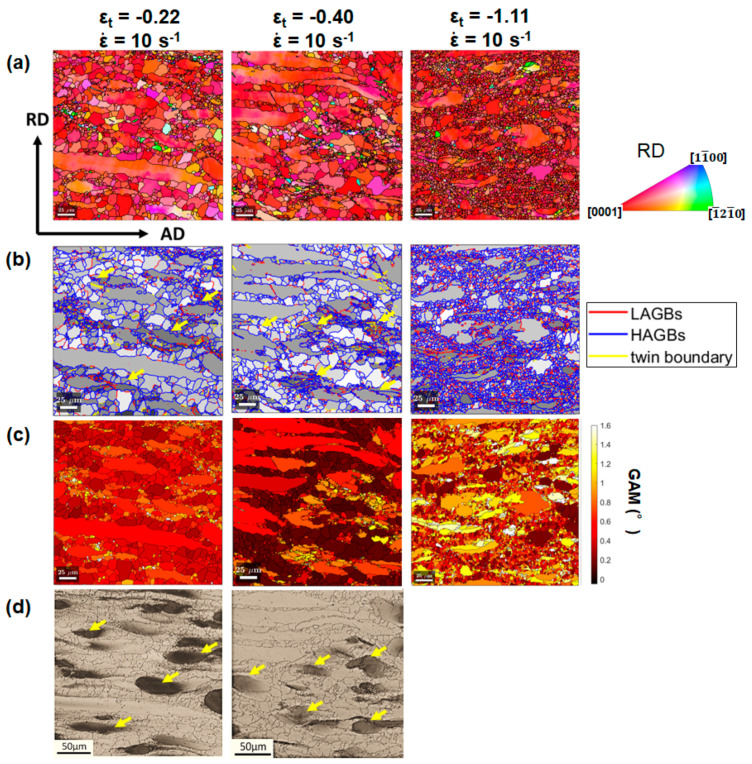
EBSD characterization of the microstructures deformed to ε_t_ of −0.22, −0.40, −1.11 at 10 s^−1^: (**a**) IPF maps and (**b**) GBC maps superimposed by grain boundaries, (**c**) GAM maps and (**d**) corresponding optical micrographs for the same ROIs. The step size for EBSD measurements was 0.3 μm. Yellow arrows indicate that dark-grey contrasted region in (**b**) correspond to Zr-rich domains in (**d**).

Region of interests (ROIs) for EBSD characterization were all from the center of the macro-SBs of the tested samples. Figure 6a shows the IPF maps for the samples deformed with ε_t_ of −0.22, −0.40, and −1.11 at $\dot{\varepsilon }$ of 10 s^−1^. Grain reconstruction for EBSD datasets was performed, based on the threshold misorientation angle of 5° between neighbor measurements. Grain boundaries were plotted as black solid lines in Figure 6a. The IPF maps confirmed the OM observation that extensive grain growth occurred at ε_t_ of −0.22 and −0.40 while profuse grain refinement occurred at ε_t_ of −1.11 for the samples hot deformed at the high strain rate, in comparison to the initial microstructure (Figure 1c).

Grain-averaged band contrast (GBC) maps superimposed with boundaries are shown in Figure 6b. Low-angle grain boundaries (LAGBs) are defined as misorientation segments in the range of 5–15°. High-angle grain boundaries are the misorientation segments >15°. Twin boundaries were identified as misorientation segments within a tolerance of 5° from the characteristic values (extension twinning of 86.3°, contraction twinning of 56°, and double twinning of 37.5°). The band contrast value reveals the quality of EBSD patterns. Higher grayscale intensity in band contrast indicates a lower quality of the EBSD pattern. Mean band contrast was calculated for each grain to plot the GBC map. Combining Figure 6b,d, it is found that Zr-rich domains corresponded to the dark-gray contrasted regions in the GBC maps. Since the quality of the EBSD pattern is significantly degraded by the lattice defects generated in plastic deformations, higher grayscale intensity in the GBC map indicates increased plastic strain [26,27]. It is thereby inferred that more deformation defects were trapped in the Zr-rich domains. It is noted that only a limited fraction of twin boundaries could be observed during the hot deformation.

Similar to GBC, grain average misorientation (GAM) has a linear correlation with the plastic strain level and is often used to quantify plastic deformation [28,29]. To begin with, the kernel average misorientation (KAM), as a measure of local misorientation [27,28,29], can be derived from EBSD measurements. For each pixel, KAM calculates the average misorientation angles between it and its neighboring pixels. Then, GAM can be derived for individual grains by averaging the KAM values of all the pixels in the grain. GAM maps in Figure 6c for the same regions in Figure 6a,b were plotted to reveal the deformation behavior of the ZK30 alloy. The grown equiaxed grains, which are lean in Zr content, showed relatively low GAM values in the microstructures. It is speculated that their occurrence is closely related to recrystallization. In addition, the fraction of domains of GAM value > 1.0° increased as deformation continued. In correlation with the corresponding OM images in Figure 6d, it seems that the domains of high GAM values roughly correspond to Zr-rich domains. Since the GAM value has a linear correlation with plastic strain, the results suggest that Zr-rich coarse grains undertook a significant proportion of the plastic strain during hot deformations.

**Figure 7:**
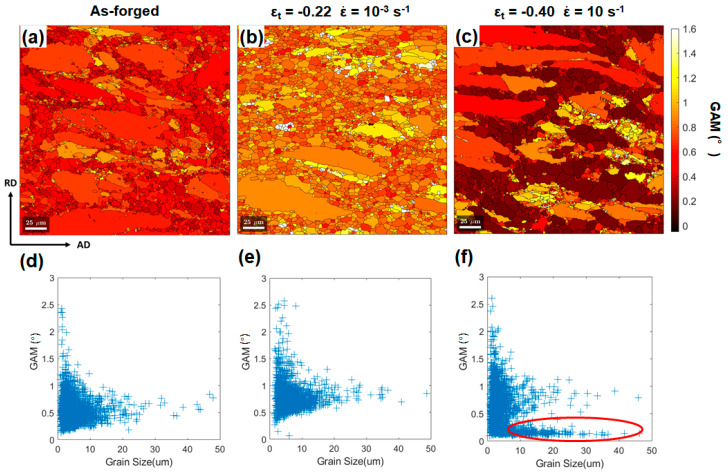
GAM maps for the microstructures in: (**a**) the as-forged state, deformed state with ε_t_ of −0.40 (**b**) at $\dot{\varepsilon }$ of 10 s^−1^ and (**c**) at $\dot{\varepsilon }$ of 10^−3^ s^−1^. The corresponding plots of grain size versus the GAM value are plotted in (**d**–**f**). The yellow ellipse in (**f**) highlights the occurrence of grown equiaxed grains with low GAM value and large grain size.

**Figure 8:**
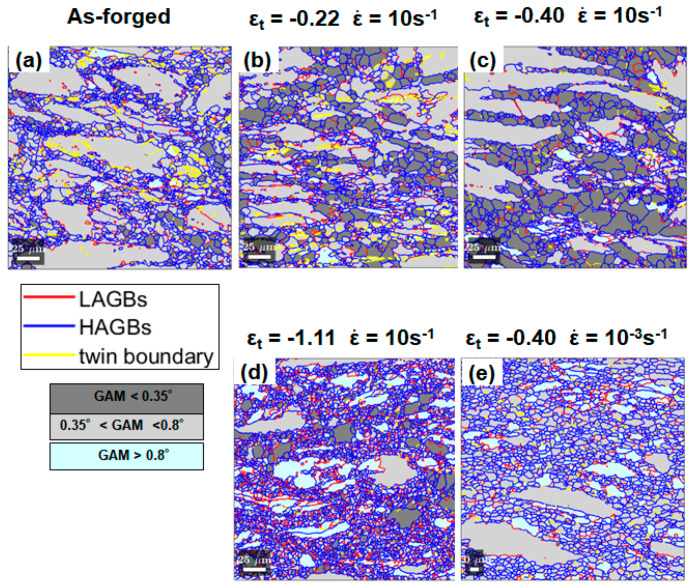
Partitioned GAM maps with threshold values of 0.35° and 0.8° for the microstructures in: (**a**) as-forged state, deformed state (**b**) with ε_t_ of −0.22 at $\dot{\varepsilon }$ of 10 s^−1^, (**c**) with ε_t_ of −0.40 at $\dot{\varepsilon }$ of 10 s^−1^, (**d**) with ε_t_ of −1.11 at $\dot{\varepsilon }$ of 10 s^−1^, and (**e**) with ε_t_ of −0.40 at $\dot{\varepsilon }$ of 10^−3^ s^−1^.

GAM maps for as-forged, high-strain-rate deformed, and low-strain-rate deformed microstructures are shown in Figure 7a–c, respectively. For a given strain level, GAM distribution seemed to be more inhomogeneous for the microstructure deformed at $\dot{\varepsilon }$ of 10 s^−1^. The corresponding plots of grain size versus the GAM value were constructed for individual conditions (Figure 7d–f). After being deformed to ε_t_ of −0.40 under the quasi-static condition, the distribution of GAM values (Figure 7e) moved toward a slightly higher value globally but retained a similar shape as that in the as-forged state (Figure 7d). However, the plot for the microstructure deformed to ε_t_ of −0.40 at $\dot{\varepsilon }$ of 10 s^−1^ (Figure 7f) shows a distinct shape owing to the occurrence of a certain fraction of grown equiaxed grains of grain size > 10 μm and low GAM values < 0.35°. The grown equiaxed grains featured by GAM value < 0.35° were visualized in dark-gray color code in the partitioned GAM maps in Figure 8.

#### 3.3.2. Texture and Misorientation Analysis of Different Groups of Grains

**Figure 9:**
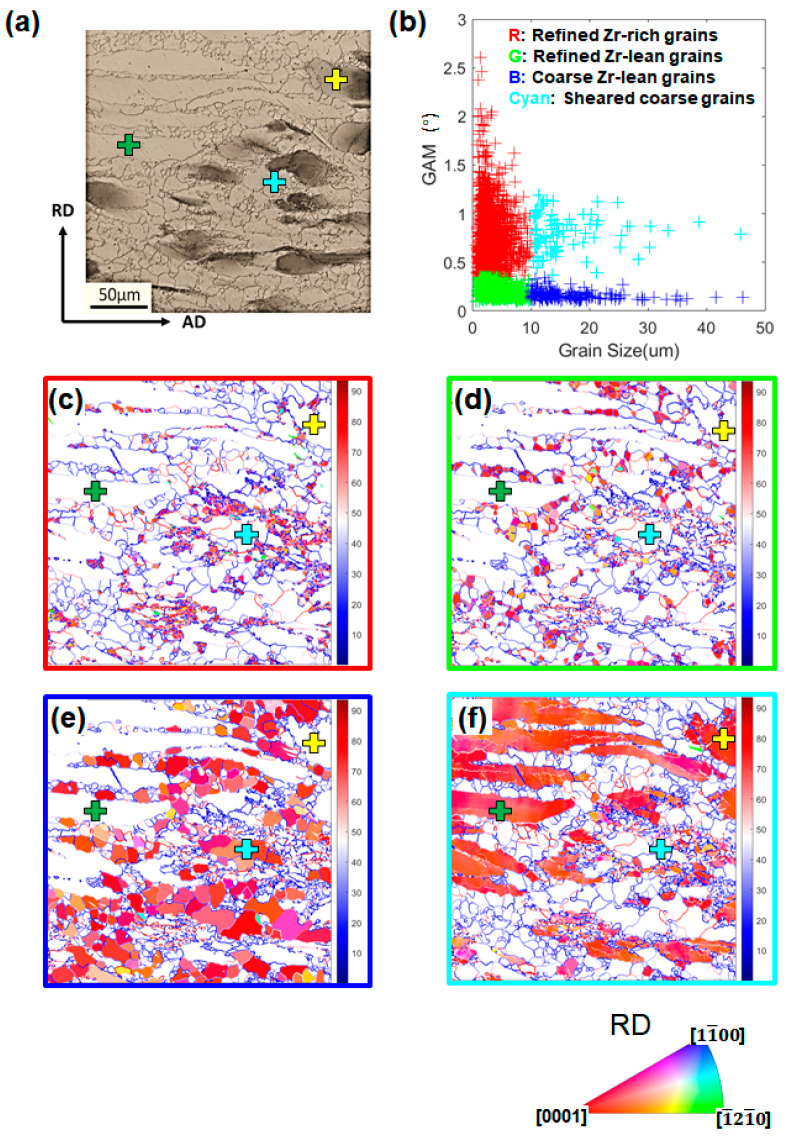
Grain partition approach based on the EBSD map for the sample deformed to a true strain of −0.69 at 10 s^−1^: (**a**) optical micrograph of the ROI, (**b**) plot of grain size versus GAM value for all the grains in the ROI. Grains were partitioned on a basis of both the GAM value (threshold value = 0.35°) and grain size (threshold value = 10 μm). Four groups are represented by red/green/blue/cyan color schemes. IPF maps for the same ROI in (a) showing (**c**) Zr-rich refined grains as the red group, (**d**) Zr-lean refined grains as the green group, (**e**) Zr-lean grown equiaxed grains as the blue group, and (**f**) sheared coarse grains as the cyan group. (The crosses on a and c–f are for the reader to position feature grains for correlation).

To further analyze the microstructural evolution during the hot compression at $\dot{\varepsilon }$ of 10 s^−1^, certain threshold values were set for the GAM value and grain size to partition grains into different groups based on correlated OM images and EBSD datasets. Figure 9 shows a typical example of this approach for the microstructure at ε_t_ of −0.40. The grains with a GAM value > 0.35° and grain size < 10 μm, represented by red symbols in Figure 9b, correspond to the refined grains with IPF color code in Figure 9c. In combination with the OM image in Figure 9a, this group of grains can be approximately referred to refined grains formed within or in the vicinity of Zr-rich coarse grains. It is thereby assumed that this group of grains resulted from the partial DRX of Zr-rich coarse grains. Grains corresponding to green symbols, in the range of the GAM value < 0.35° and grain size < 10 μm in Figure 9b, are shown in Figure 9d. They can roughly represent the fine DRXed grains lean in Zr in Figure 9a. The grown equiaxed grains in Figure 9e are mainly captured by blue symbols in the range of the GAM value < 0.35° and grain size > 10 μm. Lastly, grains represented by cyan symbols are the sheared coarse grains including both Zr-rich and Zr-lean ones, as shown in Figure 9f. Using this grain partition approach based on GAM and grain size, complicated microstructures in the deformed samples were partitioned to trace the evolution of varied groups of grains.

**Figure 10:**
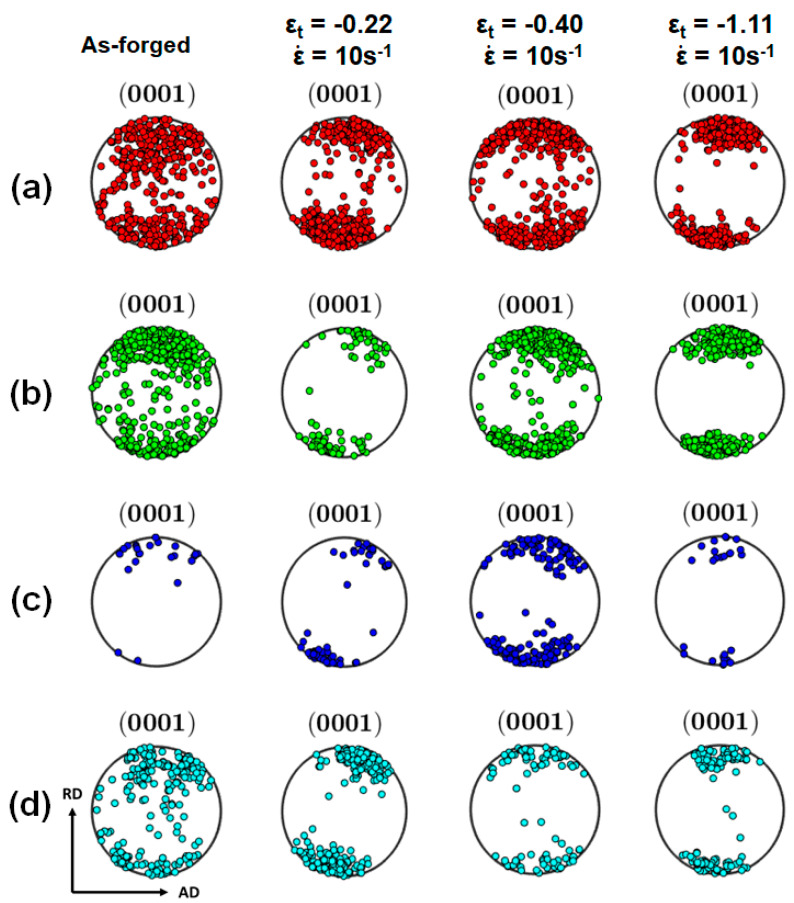
{0001} pole figures of four groups of grains: (**a**) Zr-rich refined grains as the red group, (**b**) Zr-lean refined grains as the green group, (**c**) Zr-lean grown equiaxed grains as the blue group and (**d**) sheared coarse grains as the cyan group.

The {0001} pole figures for these groups of grains are plotted in Figure 10. Figure 10d shows that the {0001} poles of the coarse grains (cyan group) in the samples hot deformed at $\dot{\varepsilon }$ of 10 s^−1^ were more concentrated to the RD than the initial coarse grains in the as-forged state. This was likely caused by the profuse operation of basal slip in the coarse grains whose c-axes initially tilted away from the RD. Figure 10b–d suggest that the refined DRXed grains (red group and green group) and the grown equiaxed grains (blue group) showed a texture similar to the sheared coarse grains after deformation. It is noted that no texture randomization occurred during the hot deformations. In general, twinning-induced DRX (TDRX) and conventional discontinuous DRX (DDRX) tended to weaken the initial texture by producing DRXed grains of randomized orientations. It is thereby inferred that TDRX and conventional DDRX were not the dominant DRX mechanisms.

**Figure 11:**
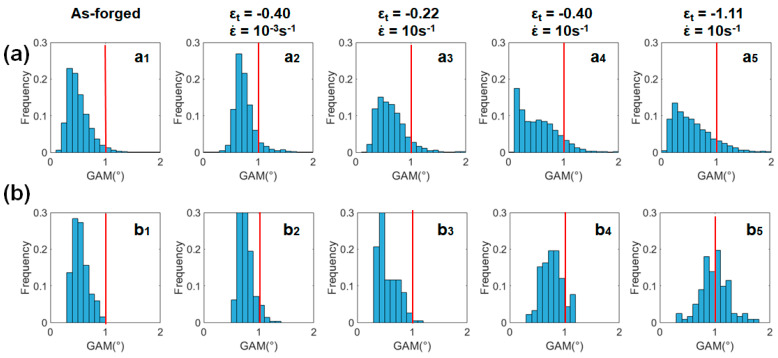
GAM histogram plots derived from EBSD data for: (**a**) all grains and (**b**) sheared coarse grains as the cyan group under varied conditions including the as-forged state, deformed states with ε_t_ of −0.22 at 10^−3^ s^−1^ and with ε_t_ of −0.22, −0.40 and −1.11 at 10 s^−1^.

Figure 11 shows the histograms for the GAM distribution of in the compressed samples, derived from Figure 6c. The sample compressed to ε_t_ of −0.44 under the quasi-static condition (Figure 11a_2_) showed a similar GAM distribution to that of the as-forged sample (Figure 11a_1_). In contrast, the fraction of grains at a high GAM value evidently increased in the high-strain-rate deformed samples, as shown in the histograms of Figure 11a_3–5_. Figure 11b_1–5_ show the GAM distribution of sheared coarse grains (cyan group in Figure 9). A comparison of Figure 11b_1_ and Figure 11b_3–5_ reveals that both the fraction of the coarse grains with a GAM value > 1.0° and the maximum GAM value increase with straining. This suggests that coarse grains gradually undertook more plastic strains with the deformation proceeding at $\dot{\varepsilon }$ of 10 s^−1^. In addition, a comparison of Figure 11b_2_ and Figure 11b_4_ indicates that for a given strain level, more plastic strains localized in coarse grains at a high strain rate. This quantitative analysis supplements and supports the microstructural observations in Figure 7 and Figure 8.

#### 3.3.3. Analysis of the Deformation/DRX Mechanisms of Zr-Rich Coarse Grains

**Figure 12:**
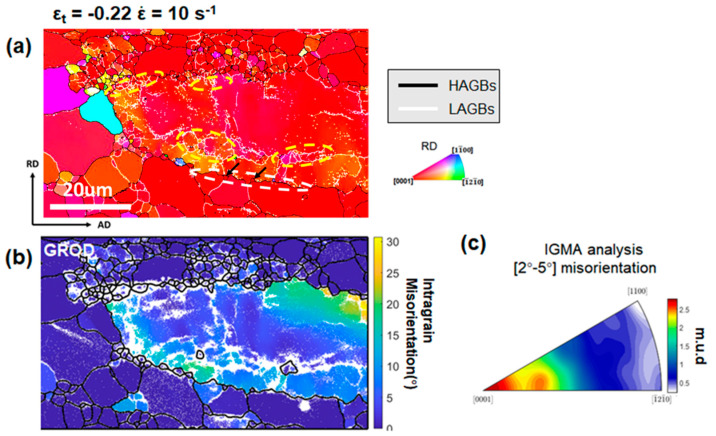
(**a**) IPF map of the microstructure deformed to ε_t_ of −0.22 at $\dot{\varepsilon }$ of 10 s^−1^ with a step size of 0.12 μm, with sub-grains marked by yellow ellipses, serrated boundaries marked by a white ellipse, and refined DRXed grains marked by black arrows. (**b**) GROD map calculated from (**a**), and (**c**) IGMA analysis of the coarse grain shown in (**b**).

Attention has been paid to Zr-rich domains for understanding the dominant DRX mechanisms operating during the hot deformations under a high-strain-rate condition. Figure 12 shows EBSD measurements with a high spatial resolution of 0.2 μm at an ε_t_ step size of −0.22 and $\dot{\varepsilon }$ of 10 s^−1^, focusing on the details of a Zr-rich coarse grain. Abundant refined sub-grains bounded by LAGBs were observed in the vicinity of the boundaries of the coarse grain, as marked by yellow ellipses in Figure 12a. With further straining, these sub-grains will be transformed into DRXed grains via progressive rotation. This is consistent with the typical CDRX mechanism reported in previous studies [12,30,31]. In addition, bulging of initial grain boundaries with serrated morphologies was observed as marked by the white ellipse in Figure 12a. Some refined grains formed along the serrated boundaries as indicated by black arrows. They may be formed by bridging the corrugated boundaries by LAGBs which can be transformed into HAGBs as the deformation continues. This DRX behavior is referred to as DDRX via grain boundary corrugation/bulging [32,33]. It is noted that the DRXed grains formed by CDRX or DDRX via grain boundary corrugation/bulging tend to have orientations similar to the parent grains [34,35]. The grain reference orientation deviation (GROD) map [29,36] for α-Mg grains is shown in Figure 12b. It is suggested that the Zr-rich coarse grain had substantial misorientations built up in the vicinity of boundaries, which supports the assumption that CDRX and DDRX were active in these regions [33]. The in-grain misorientation axes (IGMA) analysis of the coarse grain in Figure 12c revealed that the corresponding Taylor axis for the dominant slip systems activated in the coarse grain was <0001> [31,37,38,39]. The activated <0001> slip systems indicated high activity of the prismatic slip in the grain. In general, activation of non-basal slip is necessary for CDRX to be active in Mg alloys [33,40].

**Figure 13:**
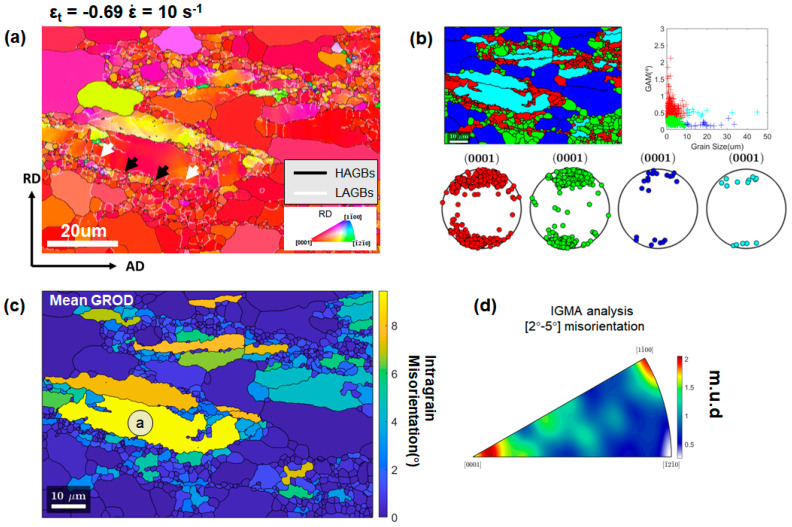
(**a**) IPF map of the microstructure deformed to ε_t_ of −0.69 at $\dot{\varepsilon }$ of 10 s^−1^ with a step size of 0.12 μm. White arrows indicate sub-grains, and black arrows indicate grain boundary corrugations. (**b**) Grain partitioning map on the basis of the GAM value and grain size. Grains were divided into a red/green/blue/cyan color scheme with the corresponding pole figures shown at the bottom, (**c**) mean GROD map, (**d**) IGMA analysis of the sheared coarse grain labeled ‘a’ in (**c**).

Figure 13a shows the IPF map superimposed with grain boundaries from an EBSD dataset at ε_t_ of −0.69 at $\dot{\varepsilon }$ of 10 s^−1^, with a step size 0.12 μm. Based on the grain partition approach elaborated in the Section 3.3.2, grains in Figure 13a were differentiated into four groups and colored in Figure 13b. Zr-rich domains, including Zr-rich coarse grains (cyan colored), and Zr-rich DRXed grains (red colored) around the coarse grains, were identified. Correlating Figure 13b with Figure 13a, sub-grains bounded by LAGBs as indicated by white arrows and grain boundary corrugations as indicated by black arrows were observed in the vicinity of the boundaries of Zr-rich coarse grains. The Zr-rich DRXed grains (red group) may have evolved from these sub-grains. This confirms that CDRX and DDRX via grain boundary corrugation/bulging were dominant in the DRX process of Zr-rich coarse grains. The corresponding mean GROD map for the same region is shown in Figure 13c. IGMA analysis (Figure 13d) of the coarse grain of the highest average GROD value, which is marked by ‘a’ in Figure 13c, indicates that the corresponding Taylor axes for the dominant slip systems activated in the coarse grain were <0001> and <$1\bar{1}00$> [31,37,38,39]. It is inferred that the co-activation of basal slip and prismatic slip contributed to the accommodation of plastic strains and the DRX process.

## 4. Discussion

### 4.1. Post-DRX (PDRX) after the Hot Compression

The growth of Zr-lean equiaxed grains occurred in the microstructures of the samples hot compressed at $\dot{\varepsilon }$ of 10 s^−1^ (Figure 4 and Figure 6). All the experimental results presented suggest that this was caused by post-DRX (PDRX) after the hot compression ceased, supported by the following:

(1) For a given strain level of −0.40, the size of the grown equiaxed grains in the sample deformed at $\dot{\varepsilon }$ of 10 s^−1^ was larger than the DRXed grains in the sample deformed at $\dot{\varepsilon }$ of 10^−3^ s^−1^ (Figure 7). In terms of a well-established theory that a higher strain rate leads to finer DRXed grains, it is unlikely that the grown equiaxed grains were produced during the high-strain-rate deformation. Moreover, the GAM values of the grown equiaxed grains at $\dot{\varepsilon }$ of 10 s^−1^ (Figure 7c) were lower than those of the DRXed grains at $\dot{\varepsilon }$ of 10^−3^ s^−1^ (Figure 7b). Therefore, it is reasonable to believe that the grown equiaxed grains were from static recrystallization (SRX) after the hot deformation ceased, owing to the quench delay in the present study. The growth of DRXed nucleus probably happened in the interval between the end of compression and water quenching (~4–5 s). Considering that DRX softening was initiated at the true strain of −0.17 (Figure 2b), the equiaxed grains with large size were more likely from the growth of the DRX nucleus produced during the hot compression. This is consistent with the phenomenon of meta-dynamic recrystallization (MDRX) [41,42,43], which is a type of PDRX. MDRX tends to be triggered during hot deformation conducted at a high strain rate. Dislocation density and stored strain energy increase with strain rate at a given temperature and for a given strain level, which provides the driving force for the growth of the DRXed nucleus [44].
(2) The fraction of the grown equiaxed grains with a GAM value < 0.35° in the high-strain-rate deformed sample at ε_t_ of −1.11 (Figure 8d) was much lower than that of the samples deformed under the same condition but at lower strains (Figure 8b,c). Stored strain energy was released via DRX in the sample deformed to ε_t_ of −1.11. Therefore, the driving force for PDRX/MDRX was significantly reduced.
(3) The grown equiaxed grains existed in the Zr-lean domains while the grains residing in the Zr-rich domains retained a refined size. The dispersion of Zn–Zr precipitates retarded grain boundary migration; thereby, the growth of the DRX nucleus formed in the Zr-rich domains was impeded. Therefore, the PDRX phenomenon could only be observed in the Zr-lean domains.

### 4.2. Microstructural Evolution of ZK30 during Hot Compression

Since the as-received material is of a bimodal microstructure from partial DRX, it is challenging to differentiate initial DRXed grains formed in forging from the DRXed grains produced in the hot compression. Moreover, the occurrence of PDRXed grains aggravates the difficulty in interpreting the microstructural evolution of the ZK30 alloy during the hot compression at $\dot{\varepsilon }$ of 10 s^−1^. 

At ε_t_ of −0.22, the DRX softening outweighed strain hardening as reflected by the decline of true stress beyond the peak in the corresponding stress–strain curve (Figure 2b). At this strain level, the plastic strain undertaken by initial coarse grains was not significant, and deformation was not concentrated to macro-SBs (Figure 2c and Figure 4b). The DRXed nucleus probably first formed in the Zr-lean regions during the compression and grew into the matrix of highly stored strain energy after the compression ceased. It is noted that the grown equiaxed grains from PDRX roughly inherited the orientation of the matrix, referring to Figure 10. It is inferred that the original DRXed nucleus had similar orientations as its parent grain.

With the deformation proceeding to ε_t_ of −0.40, strain was localized to macro-SBs (Figure 3). This is supported by Figure 11b showing that the GAM values of coarse grains in the samples deformed at $\dot{\varepsilon }$ of 10 s^−1^ underwent a marked increment with increased strain. Meanwhile, more Zr-rich refined DRXed grains formed near the coarse grains at ε_t_ of −0.40 (Figure 4c). The Zr-rich DRXed refined grains were produced via CDRX and DDRX via boundary bulging along the boundaries of coarse grains, as elaborated in the Section 3.3.3. In the latter stage of hot compressions at ε_t_ of −0.69 and −1.11, more stored strain energy was released by profuse DRX. Therefore, the fraction of PDRXed grains in the microstructure at ε_t_ of −1.11 was much lower than that in the microstructures at lower strain levels (Figure 8). In addition, coarse grains were gradually consumed by DRXed grains with straining (Figure 5).

### 4.3. Plasticity of ZK30 during Hot Compression at $\dot{\varepsilon }$ of 10 s^−1^

Although TDRX was suppressed in the as-forged ZK30 alloy in the hot compression at $\dot{\varepsilon }$ of 10 s^−1^, the ZK30 alloy still exhibited excellent plasticity in the present study. This can be attributed to the following:

(1) Severe shear banding related to geometrical softening caused by the formation of compression twinning and double twinning in the basal-textured matrix was impeded. This is because twinning was suppressed due to the refined grain size and high testing temperature.
(2) The dominant DRX mechanisms operating in the present study were CDRX and DDRX via boundary corrugation (Figure 12 and Figure 13). DRXed grains produced via these mechanisms tended to have similar orientations as their parent grains. Although they coalesced to form a necklace structure around the coarse grains, strains were not concentrated in the necklace structure to form shear bands. This is because the DRXed grains in the necklace structure had similar Schmid factors for slip as the parent grains.
(3) It is believed that Zr-rich coarse grains played an essential role in accommodating plastic strains during the hot compression at $\dot{\varepsilon }$ of 10 s^−1^. It was observed that coarse grains in the macro-SBs gradually undertook more plastic strain with straining. Since the distribution of Zr-rich coarse grains was relatively homogeneous in the macro-SBs, deformation within the macro-SBs was homogenized and thereby, good plasticity of the as-forged ZK30 alloy was achieved. Referring to Figure 12 and Figure 13, prismatic slip was activated in the Zr-rich coarse grains. Non-basal slip activity contributed to the operation of CDRX in the vicinity of Zr-rich coarse grains, leading to the relaxation of stress concentration at the interphase. In addition, the activated non-basal slip enhanced the plasticity of Zr-rich coarse grains by providing more independent deformation mechanisms.

**Figure 14:**
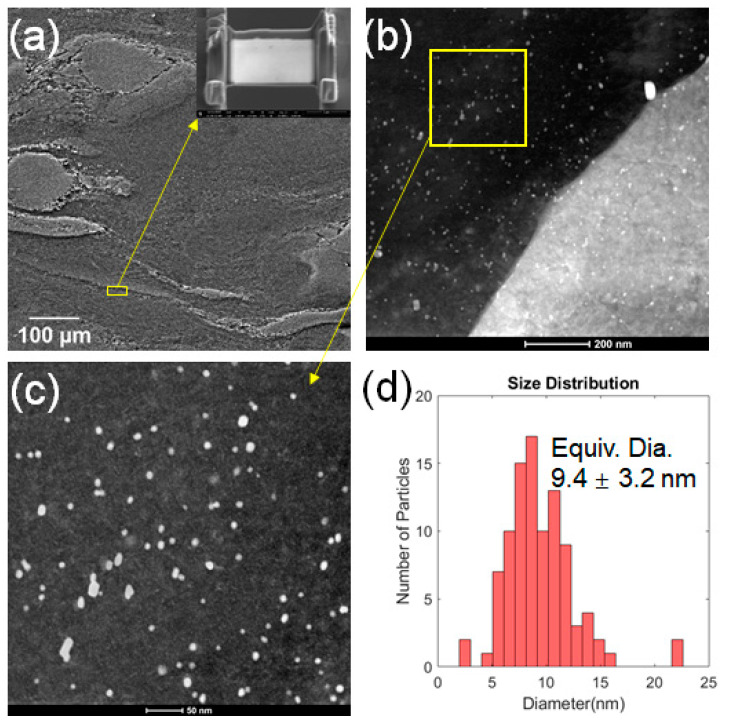
(**a**) SEM image of the selected area for FIB life-out membrane from the sample deformed to ε_t_ of −0.40 at $\dot{\varepsilon }$ of 10 s^−1^, (**b**) HAADF image in STEM mode for the sample lifted out in (**a**), (**c**) nano-sized Zn–Zr particles, and (**d**) size distribution histogram quantified from the micrograph in (**c**) showing the mean equivalent diameter of these Zn–Zr particles was about 9.4 nm.

The high activity of prismatic slip in the Zr-rich coarse grains may be correlated with the reduction of the ratio of critical resolved shear stress (CRSS) of the prismatic slip to that of the basal slip. The reduced deformation anisotropy is believed to be caused by the dispersion of Zn–Zr precipitates within the coarse grains. As shown in Figure 14 obtained from the sample deformed to ε_t_ of −0.40 at $\dot{\varepsilon }$ of 10 s^−1^, Zn–Zr precipitates observed in the Zr-rich domains exhibited spherical morphology with the average equivalent diameter of ~9.4 nm and showed a high number density. It is generally thought that Zn–Zr particles dispersed in the matrix of Mg–Zn–Zr alloys do not have a marked contribution to the overall strength since they only form in a limited number of coarse grains with Zr segregation [45]. However, when it comes to individual coarse grains enriched in Zn–Zr particles, the strengthening effect of the precipitates on basal and prismatic slips may be noticeable. Given that the strengthening effect of Zn–Zr precipitates on the CRSS of basal and prismatic slips are *∆τ_basal_* and *∆τ_prism_*, the ratio *CRSS_prim_*/*CRSS_Basal_* will change to (*CRSS_prim_* + *∆τ_prism_*)/(*CRSS_Basal_* + *∆τ_basal_*) when the effect of Zn–Zr precipitates is accounted for. If *∆τ_prism_*/*∆τ_basal_* < *CRSS_prim_*/*CRSS_Basal_*, the ratio will be reduced, rendering prismatic slip more readily active. The ratio *CRSS_prim_*/*CRSS_Basal_* of Mg alloys normally ranges from ~2.5–7 [46,47]. Considering that the Zn–Zr particles observed in this study showed spherical morphology, their strengthening effects on basal slip and prismatic slip is likely to be similar based on the Orowan bowing mechanism [48]. Therefore, it is reasonable to assume that the dispersion of Zn–Zr precipitates in Zr-rich coarse grains will reduce the ratio *CRSS_prim_*/*CRSS_Basal_*, leading to high activity of prismatic slip in the grains. Similar effects of precipitated (Mg,Al)_2_Ca particles in AZ31−0.5Ca alloy and G.P. zones in Mg–Al–Ca–Mn alloys on the reduced deformation anisotropy and enhanced plasticity of Mg alloys have been reported [49,50,51]. This study reveals that enhanced activity of non-basal slip is essential for the excellent plasticity of Mg alloys during deformation under a high-strain-rate condition.

## 5. Conclusions

The present study investigates the microstructural evolution of an as-forged ZK30 alloy during uniaxial hot deformation at 350 °C at a high strain rate of 10 s^−1^. The complicated deformed microstructures were successfully separated into distinct groups of grains. Correlative imaging and a grain partitioning approach based on intra-granular misorientation analysis of EBSD measurements were used. Focus was placed on the deformation and DRX behaviours of the alloy. The following conclusions can be made from this work:

1. The PDRX phenomenon was observed in the Zr-lean regions of the ZK30 samples hot deformed to true strains ranging from −0.22 to −0.69 at a strain rate of 10 s^−1^. The experimental results suggested that the DRXed nucleus produced during the hot compression grew into the matrix of high stored strain energy after the deformation ceased. However, for the sample deformed to a true strain of −1.11 at the same strain rate, stored strain energy was significantly released by profuse DRX and hence, PDRX behavior was impeded.
2. A true strain of −1.11 can be achieved during the hot compression of the as-forged ZK30 alloy at a temperature of 350 °C and a strain rate of 10 s^−1^, without any cracks observed. The alloy exhibited excellent formability under the present deformation condition.
3. During the hot deformation at a strain rate of 10 s^−1^, a macro-shear band started to form at a true strain of −0.22, and plastic strains localized to it with the deformation continued. Within the shear band, a ‘necklace structure’ consisting of refined DRXed grains was formed around the Zr-rich coarse grains. CDRX and DDRX via grain boundary corrugation/bulging were the dominant recrystallization mechanisms. As the deformation continued, the fraction of refined DRXed grains in the Zr-rich domains increased and the aspect ratio of coarse grains dramatically increased. In contrast, the microstructure after hot compression at a strain rate of 10^−3^ s^−1^ under the same temperature was much more uniform for the same strain level.
4. Zr-rich coarse grains, which were homogeneously distributed in the as-received microstructure, played an important role in accommodating plastic strains in the hot deformation at a high strain rate. It is speculated that the densely distributed Zn–Zr particles of refined size reduced the ratio of the critical resolved shear stress of the prismatic slip to that of the basal slip. This contributed to the activation of prismatic slips enhancing the plasticity of coarse grains. Moreover, the high activity of prismatic slips facilitated the operation of CDRX in the vicinity of grain boundaries, which effectively relaxed the stress concentration.
5. The present study suggests that the twinning-induced DRX mechanism is not necessary for the excellent plasticity of Mg alloys in hot deformation under a high-strain-rate condition. Enhanced activity of non-basal slips appears to be more essential to improve the plasticity of Mg alloys hot deformed at a high strain rate.
